# Efficacy of Omeprazole, Tetracycline, and 4 Times Daily Dosing of Amoxicillin in *Helicobacter pylori* Eradication in Limited Resource Area in Bhutan: A Prospective Randomized Trial (BHUTAN Study)

**DOI:** 10.31557/APJCP.2020.21.4.1109

**Published:** 2020-04

**Authors:** Ratha-Korn Vilaichone, Natsuda Aumpan, Thawee Ratanachu-ek, Pornpen Gumnarai, Tomohisa Uchid, Lotay Tshering, Varocha Mahachai, Yoshio Yamaoka

**Affiliations:** 1 *Gastroenterology Unit, Department of Medicine, Faculty of Medicine, Thammasat University Hospital, *; 2 *Department of Medicine, Chulabhorn International College of Medicine (CICM), *; 3 *Digestive diseases Research Center (DRC), *; 5 *Department of Biochemistry, Faculty of Medicine, Thammasat University, Pathumthani, *; 4 *Department of Surgery, Rajavithi Hospital, *; 8 *Gastrointestinal and Liver Center, Bangkok Medical Center, Bangkok, Thailand, *; 6 *Department of Molecular Pathology, *; 9 *Department of Environmental and Preventive Medicine,Oita University Faculty of Medicine, Yufu, Japan, *; 7 *Department of Surgery, Jigme Dorji Wangchuk National Referral Hospital, Thimphu, Bhutan. *

**Keywords:** Helicobacter pylori eradication, limited resource area, Bhutan

## Abstract

**Backgrourd:**

*H. pylori*-associated gastric cancer is the first cancer-related death in Bhutan. Effective regimen for *H. pylori *eradication is essential to reduce risk of developing gastric cancer. Clarithromycin is not widely used in this limited resource country. Aim of this study was to evaluate proper regimen and prevalence of antibiotic resistance pattern for *H. pylori *eradication in Bhutan.

**Methods:**

Five hundred and forty-six patients underwent gastroscopy during GASTROCAMP between October 2014 and April 2015 in Bhutan and 77 patients were enrolled. Four gastric biopsies were obtained for rapid urease test, histopathology, *H. pylori* culture with Epsilometer test. All *H. pylori-*positive patients were randomized to receive either 7-day or 14-day of 500 mg amoxicillin four times daily, 500 mg tetracycline four times daily, and 20 mg omeprazole twice daily.

**Results:**

Seventy-seven subjects were enrolled (54 females, 23 males, mean age = 45.4 years). Of 77 patients, 52 (67.5%) received 7-day regimen while 25 (32.5%) had 14-day regimen. Prevalence of *H. pylori* was 38.2%. Antibiotic resistance was 80.0% for metronidazole, 11.1% for levofloxacin and no resistance seen in amoxicillin, tetracycline and clarithromycin. Overall eradication rates of 7-day and 14-day regimens were 51.9% and 80.0%, p = 0.02. Female and age ≥40 years had significantly higher eradication rate when receiving 14-day compared to 7-day regimen (94.1% vs. 45.9%, OR = 18.82; 95% CI 2.26-157.02, p = 0.0007 and 86.7% vs. 50.0%, OR = 6.50; 95% CI 1.25-33.91, p = 0.02, respectively).

**Conclusions:**

Fourteen-day regimen might be an acceptable regimen for *H. pylori* eradication in limited resource area such as Bhutan. Female and age ≥40 years should receive longer duration of treatment. This 14-day regimen could at least reduce the risk of developing *H. pylori*-associated diseases especially peptic ulcer with complications and gastric cancer which lead to many deaths in Bhutan.

## Introduction

*Helicobacter pylori* (*H. pylori*) is a gram-negative bacterium which colonizes and causes chronic infection of stomach (Dunn et al., 1997). Milder form of *H. pylori* infection is gastric mucosal inflammation or gastritis. However, it can eventually lead to more serious conditions such as gastric mucosa–associated lymphoid tissue (MALT) lymphoma, and gastric cancer, which is the third leading cause of cancer deaths according to global cancer statistics in 2018 (McColl, 2010; Srinarong et al., 2014; Vilaichone et al., 2014; Bray et al., 2018; Vilaichone et al., 2018; Poonyam et al., 2019). Host genetic polymorphisms regulating inflammatory response to *H. pylori* infection, along with bacterial virulence and colonization factors play an important role in the pathogenesis of *H. pylori-*related disease (Amieva and El-Omar, 2008). Therefore, host, bacteria and environment integration are factors exerting influence on distinct clinical outcomes between each location (Kuster et al., 2006; Yamaoka and Graham, 2014). Compared to other regions, Asia has the highest incidence and prevalence of gastric cancer accentuating potential health concerns in this area (Rahman et al., 2014).

Kingdom of Bhutan is a landlocked country located in South Asia. Bhutan is bordered by Tibet to its north and west, while India borders its west, east, and south. Considered as one of the countries with the lowest population density, Bhutan has total population of 735,553 per total area of 38,394 square kilometers. The country’s geographical features mainly comprise deep valleys and steep mountains with most Himalayan peaks in the north elevated over 7,000 meters above sea level. In this country, cancer is responsible for 10% of all deaths with gastric cancer being the leading cause of cancer mortality. In Bhutan, stomach cancer had the highest incidence of 20.7%, and mortality rate of 23.9% making it the most important disease to focus on (World Health Organization, 2018). Moreover, Bhutan’s age-standardized mortality rate of gastric cancer was relatively high as 18.9 per 100,000 compared to 8.2 of global range (International Agency for Research on Cancer, 2018). Persistent *H. pylori* infection is a major risk factor of developing non-cardia gastric cancer (Plummer et al., 2015). The overall prevalence of *H. pylori *infection in Bhutan population was extremely high as 73.4% which could contribute to high gastric cancer incidence in Bhutan (Vilaichone et al., 2013). Therefore, effective *H. pylori* eradication should be taken into consideration for gastric cancer prevention (Mahachai et al., 2016). However, the information about eradication therapy in Bhutan is still lacking. 

Until now, the issue about *H. pylori *treatment in Bhutan has not been addressed before. The purpose of this study was to evaluate proper duration of antibiotic treatment for *H. pylori*-infected patients in order to implement the most effective regimen for *H. pylori *eradication in Bhutan population. In this prospective randomized trial, we used triple therapy for 7 or 14 days to eradicate the bacteria. The antimicrobial susceptibility testing was also performed to determine the distribution of antibiotic resistant *H. pylori* strains in Bhutan.

## Materials and Methods

*Patients*

Five hundred and forty-six patients underwent upper GI endoscopy during GASTROCAMP between October 2014 and April 2015 in Bumthang and Haa provinces, Bhutan. A total of 77 patients with dyspepsia residing in the cities named Bumthang and Haa were enrolled in this prospective study. There were 23 males and 54 females with the mean age of 45.4 years. All patients included in this study underwent upper GI endoscopy and were diagnosed with non-ulcer dyspepsia which was defined as normal or mild gastritis. We excluded patients receiving H2 receptor antagonists, proton pump inhibitor (PPI), bismuth compound, and antimicrobial agents within 4 weeks prior to the study, using NSAIDs and anticoagulant, having history of stomach surgery, or having significant comorbidities such as renal failure, advanced cirrhosis, advanced-stage cancer, or cardiac arrhythmia. Special population such as immunocompromised hosts, pregnant women, or breastfeeding women were also excluded. Informed consent was obtained from volunteers at the beginning of the study. 

*Sample collection*

All 77 Bhutanese patients in the study underwent upper GI endoscopy and 4 gastric biopsies were obtained for rapid urease test, histopathology, *H. pylori* culture with antimicrobial susceptibility tests (Epsilometer test). 

*Rapid urease test*

The test label was peeled off and an antral biopsy was placed in the center of the test well. Then, the test label was resealed and left at room temperature for 60 minutes. If there were *H. pylori* in the tissue specimen, the bacterial urease would convert urea to ammonia and consequently change the pH indicator color from yellow to pink. In this study, all patients with positive rapid urease test were given triple therapy.

*Histopathology*

The gastric biopsies were processed in the embedding and cutting procedure. They were later stained with hematoxylin and eosin. A positive *H. pylori *biopsy sample was defined as a sample with curved rod-shaped bacteria.

*H. pylori culture*

The Eppendorf tubes containing transport media were used for antral biopsy collection. One antral biopsy was minced and mixed in the broth and then streaked on a Mueller Hinton - Agar medium using a sterile heated wire loop. The medium was then put in the candle jar and incubated at 37°C in microaerophilic condition for 3 to 5 days. Colors of *H. pylori* colonies ranged from translucent to pale grey on blood agar. For *H. pylori *detection, gram staining was used and showed small curved gram-negative, rod-shaped bacteria. The biochemical tests including oxidase, catalase, and urease tests were all positive for *H. pylori.*

*Antimicrobial susceptibility testing*

The Epsilometer test (E-test) provides information about antimicrobial susceptibility and determines the minimum inhibitory concentrations (MICs) of antibiotics including amoxicillin (AMX), clarithromycin (CLR), metronidazole (MNZ), tetracycline (TET), and levofloxacin (LVX). The bacteria were inoculated on the plates on which E-test strips with antibacterial agents were placed. Three to five days later, an ellipse became visible. The MIC was defined by the point of intersection between an ellipse and the MIC reading scale which indicated the lowest concentration of antibiotics that was able to inhibit visible bacterial growth. Resistant strains were considered when MIC values were ≥ 0.25 μg/mL for AMX, ≥ 1 μg/mL for CLR, ≥ 8 μg/mL for MNZ, ≥ 1 μg/mL for LVX, and ≥ 4 μg/mL for TET (Mégraud F and Lehours, 2007). 

*Therapeutic regimens*

All patients with positive rapid urease test were randomized into 2 groups using a computer-generated list of random numbers to receive either 7-day or 14-day of 500 mg amoxicillin four times daily, 500 mg tetracycline four times daily, and 20 mg omeprazole twice daily.

*Post-therapy follow-up*

*H. pylori* eradication was confirmed by using 13C- urea breath test (13C-UBT) at 4 weeks after completing a course of the triple therapy. 13C-UBT is a non-invasive test determining treatment success by detection of labeled CO_2_ exhaled in a breath sample. If the result showed negative 13C-UBT then the patient was considered achieving successful *H. pylori* eradication (Tongtawee et al., 2014).

*Statistical analysis*

The statistical analysis was performed by using SPSS version 22 (SPSS Inc., Chicago, IL, USA). The demographic data were analysed by unpaired t-test, Fisher’s exact test, and Chi-square test where appropriate. Statistical significance was defined as a two-tailed p-value cut point of less than 0.05.

## Results

Total of 77 patients were enrolled in the study. The mean age of all patients was 45.4 years with female preponderance (70.1%). Of 77 patients, 52 (67.5%) received 7-day regimen while 25 (32.5%) had 14-day regimen. The majority of patients came from Haa (74.0%), the district in the west of Bhutan while the rest were from Bumthang (26.0%), the district in central region. Every patient in the study had* H. pylori* infection identified by positive rapid urease test and was given either 7-day or 14-day triple therapy. The endoscopic findings of all patients were gastritis. Demographic data including gender, age group, and the study location were demonstrated in Table 1.

**Figure 1:**
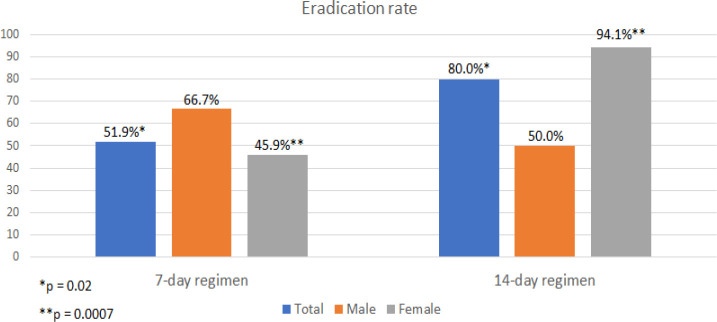
Effect of Genders on *H. pylori* Eradication Rate

**Figure 2.**
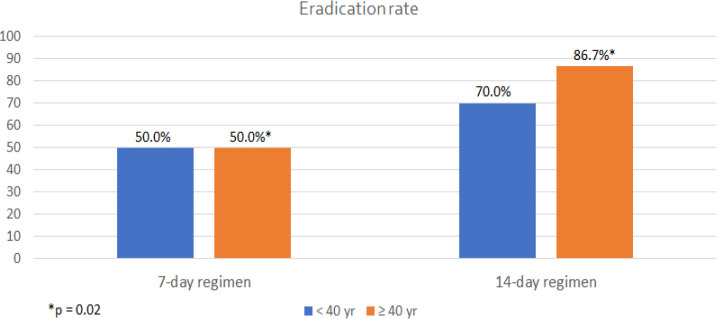
Effect of Age Groups on *H. pylori* Eradication Rate

The *H. pylori* eradication rate of 7-day triple therapy was significantly lower than that of 14-day regimen (51.9% vs. 80.0%, p = 0.02). Classified by gender, 37 of all 54 females received 7-day regimen while 17 received 14-day treatment. However, males in both groups showed relatively low eradication rate with only 66.7% and 50% in 7-day and 14-day triple therapy, respectively. When categorized into two age groups, the successful eradication was observed remarkably higher in patients older than 40 years receiving 14-day therapy compared to 7-day treatment (86.7% vs. 50.0%; p = 0.02). The younger-than-40-year age group also demonstrated the increase in eradication rate from 50% in 7-day therapy to 70% in 14-day therapy but could not reach statistical significance (p = 0.44). Patients receiving 14-day therapy were more likely to have successful eradication than the group receiving 7-day regimen (OR = 3.70; 95%CI 1.21-11.36, p = 0.02). Female and age ≥40 years subgroup also demonstrated significantly higher eradication rate when receiving 14-day compared to 7-day regimen (94.1% vs. 45.9%, OR = 18.82; 95% CI 2.26-157.02, p = 0.0007 and 86.7% vs. 50.0%, OR = 6.50; 95% CI 1.25-33.91, p = 0.02, respectively). The eradication rate and factors affecting on the treatment outcome were shown in Table 2. The difference of eradication rate classified by gender and age group was demonstrated in Figure 1 and 2, respectively.

The gastric biopsies of patients with positive urease test were sent for *H. pylori* culture. Forty-five patients had positive culture (78.9%). Thirty-six patients (80.0%) in the study mainly had metronidazole-resistant strains. Five patients (11.1%) were infected with strains resistant to metronidazole and levofloxacin. Interestingly, no resistance seen in amoxicillin, tetracycline and clarithromycin. There was no difference of antibiotic-resistant strains between genders. The antibiotic susceptibility testing was demonstrated in Table 3. 

**Table 1:** Demographic Data

Demography	Total (n = 77)	7-day regimen (n = 52)	14-day regimen (n = 25)	*P-*value
Gender				
Men	23 (29.9%)	15 (28.8%)	8 (32.0%)	0.78
Women	54 (70.1%)	37 (71.2%)	17 (68.0%)	0.78
Mean age ± SD (years)	45.4 ± 12.7	45.8 ± 13.8	44.7 ± 10.2	0.74
Range	17 – 75	17 – 75	30 – 61	
< 40 years	30 (40.0%)	20 (40.0%)	10 (40.0%)	1
≥ 40 years	45 (60.0%)	30 (60.0%)	15 (60.0%)	1

**Table 2:** Effect of Clinical Factors on *H. pylori* Eradication Rate

Factors	7-day regimen	14-day regimen	p-value
Total	27/52 (51.9%)	20/25 (80.0%)	0.02
Gender			
Men	10/15 (66.7%)	4/8 (50.0%)	0.66
Women	17/37 (45.9%)	16/17 (94.1%)	0.0007
Age			
< 40 years	10/20 (50.0%)	7/10 (70.0%)	0.44
≥ 40 years	15/30 (50.0%)	13/15 (86.7%)	0.02

**Table 3:** Antibiotic Susceptibility Testing

Antibiotics	Total (n = 45)	Male (n = 19)	Female (n = 26)	*P*-value
Antibiotic resistance				
-Amoxicillin (AMX)	0	0	0	-
-Clarithromycin (CLR)	0	0	0	-
-Metronidazole (MNZ)	36 (80.0%)	15 (78.9%)	21 (80.8%)	1.00
-Tetracycline (TET)	0	0	0	-
-Levofloxacin (LVX)	5 (11.1%)	2 (10.5%)	3 (11.5%)	1.00
-MNZ and LVX	5 (11.1%)	2 (10.5%)	3 (11.5%)	1.00

## Discussion

Bhutan is a small peaceful sovereign state in South Asia located on the eastern edge of the Himalayas. Its landscape ranges from tropical plains in the south to Himalayan forests and mountains in the north. Although the country has been opened to foreigners since 1974, there are still low numbers of international tourists entering the kingdom. In medical field, there has also been a small number of clinical research conducted recently. As cancer incidence in Bhutan has been rising, Bhutan cancer society was founded in 2015 in order to raise awareness of cancer prevention and research. Gastric cancer is the most prevalent cancer in Bhutan and often diagnosed at an advanced stage (Dendup et al., 2015). Chronic *H. pylori* infection was proved to be associated with gastric cancer in many studies (Parsonnet et al., 1991; Vilaichone et al., 2006; Karami et al., 2013). The previous study revealed that the overall prevalence of *H. pylori* infection in Bhutanese population was as high as 73.4% and the rate was even higher in rural areas (Vilaichone et al., 2013). Our study was conducted in rural districts named Haa and Bumthang in which we supposed that the prevalence would be as high as 80-90% as mentioned in two previous studies (Vilaichone et al., 2013; Dorji et al., 2014). The study population was predominantly female (70.1%) and the mean age was 45.4 years which were slightly higher than proportion of females (57.5%) and the mean age (39.6 years) in the prior study (Vilaichone et al., 2013). In our study, all patients with positive urease test were prescribed triple therapy composed of amoxicillin, tetracycline, and omeprazole and scheduled a follow-up visit. Tetracycline was used instead of clarithromycin because tetracycline had a local stock while clarithromycin was not available and had to be imported from India. 

Our study demonstrated that 14-day triple therapy had significantly higher *H. pylori* eradication rate (80%) than 7-day regimen (51.9%). In concordance with previous studies and systematic reviews, the duration of triple therapy should be extended to 14 days to improve eradication rate (Yuan et al., 2013; Mahachai et al., 2016). The higher eradication rate of 14-day regimen was also significant in female than male subgroup which might be explained by more severe mucosal inflammation and activity resulting from different level of intramucosal cytokines and genetic expression in men (Kato et al., 2004). Classified by age group, the *H. pylori* eradication rate in older age group was superior to that of the younger generation and significantly improved by using 14-day triple therapy. The previous study in Japan revealed that people in older age group were inclined to achieve more successful eradication because of more prevalent gastric atrophy and acid hyposecretion leading to superior antibiotic response (Mamori et al., 2010). Apart from host factors, bacterial factors such as virulence factor and antibiotic resistance also play important roles in *H. pylori* treatment. 

The antimicrobial susceptibility of Bhutanese patients in this study exhibited the strains mostly resistant to metronidazole (80.0%). The metronidazole-resistant rate was approximately the same as 82.9% of previous study conducted in districts of Thimphu, Punaka, and Wangdue of Bhutan (Vilaichone et al., 2013). However, the multidrug-resistant strains (11.1%) were more frequently found in our study than our prior report (2.7%) (Vilaichone et al., 2013). Multidrug-resistant strains isolated in both studies showed resistance to the same types of antibiotics which were metronidazole and fluoroquinolones. Rising numbers of multidrug-resistant strains may be due to increasing antibiotic use. Bhutan’s communicable, maternal, perinatal, and nutritional conditions were responsible for 21% of premature deaths which were three times higher than the rate of developed countries. Metronidazole is commonly used for treatment of diarrheal diseases, gynecologic and periodontal infections, whereas levofloxacin is generally used for lower respiratory tract infection. Both gastrointestinal and respiratory infection are major problems in the country and can probably cause an increasing rate of the two most common antibiotic resistance in Bhutan. It can be concluded that metronidazole-based triple therapy should not be used as first-line regimen in the country with high primary metronidazole resistance (Katelaris, 2009). Nevertheless, the previous study in Thailand showed that bismuth-based quadruple therapy containing metronidazole could still be used despite high metronidazole resistance (Vilaichone et al., 2015). Moreover, levofloxacin-based regimen may also have reduced efficacy and cannot be used as a substitute regarding the growing resistant strains to fluoroquinolone (Perna et al., 2007). Since no resistance to amoxicillin, tetracycline, and clarithromycin was noted in our study, these antibiotics can still be used in the first-line treatment for *H. pylori* in Bhutan.

Gastric cancer ranked highest in prevalence, incidence and cancer mortality in Bhutan. Our study suggested that 14-day omeprazole, tetracycline, and 4 times daily dosing of amoxicillin might be an acceptable regimen for *H. pylori* eradication in limited resource area such as Bhutan. This regimen could reduce the risk of developing *H. pylori*-associated diseases especially peptic ulcer with complications and gastric cancer leading to many deaths in Bhutan. 
